# Risk and prognosis of *Staphylococcus aureus* bacteremia among individuals with and without end-stage renal disease: a Danish, population-based cohort study

**DOI:** 10.1186/s12879-014-0740-8

**Published:** 2015-01-08

**Authors:** Lise H Nielsen, Søren Jensen-Fangel, Thomas Benfield, Robert Skov, Bente Jespersen, Anders R Larsen, Lars Østergaard, Henrik Støvring, Henrik C Schønheyder, Ole S Søgaard

**Affiliations:** Department of Infectious Diseases, Aarhus University Hospital, Brendstrupgaardvej 100, 8200 Aarhus N, Denmark; Department of Infectious Diseases, Hvidovre Hospital, University of Copenhagen, Copenhagen, Denmark; Department of Clinical Medicine, Faculty of Health and Medical Sciences, University of Copenhagen, Copenhagen, Denmark; Statens Serum Institut, Hillerød, Denmark; Department of Nephrology, Aarhus University Hospital, Aarhus N, Denmark; Department of Public Health, Biostatistics, Aarhus University, Aarhus N, Denmark; Department of Clinical Microbiology, Aalborg University Hospital, Aalborg, Denmark

**Keywords:** Bacteremia, Staphylococcus aureus, End-stage renal disease, Dialysis

## Abstract

**Background:**

*Staphylococcus aureus* is a leading cause of bloodstream infections among hemodialysis patients and of exit-site infections among peritoneal dialysis patients. However, the risk and prognosis of *Staphylococcus aureus* bacteremia among end-stage renal disease patients have not been delineated.

**Methods:**

In this Danish nationwide, population-based cohort study patients with end-stage renal disease and matched population controls were observed from end-stage renal disease diagnosis/sampling until first episode of *Staphylococcus aureus* bacteremia, death, or end of study period. *Staphylococcus aureus* positive blood cultures, hospitalization, comorbidity, and case fatality were obtained from nationwide microbiological, clinical, and administrative databases. Incidence rates and risk factors were assessed by regression analysis.

**Results:**

The incidence rate of *Staphylococcus aureus* bacteremia was very high for end-stage renal disease patients (35.7 per 1,000 person-years; 95% CI, 33.8-37.6) compared to population controls (0.5 per 1,000 person-years; 95% CI, 0.5-0.6), yielding a relative risk of 65.1 (95% CI, 59.6-71.2) which fell to 28.6 (95% CI, 23.3-35.3) after adjustment for sex, age, and comorbidity. After stratification for type of renal replacement therapy, we found the highest incidence rate of *Staphylococcus aureus* bacteremia among hemodialysis patients (46.3 per 1,000 person-years) compared to peritoneal dialysis patients (22.0 per 1,000 person-years) and renal transplant recipients (8.9 per 1,000 person-years). In persons with *Staphylococcus aureus* bacteremia, ninety-day case fatality was 18.2% (95% CI, 16.2%-20.3%) for end-stage renal disease patients and 33.7% (95% CI, 30.3-37.3) for population controls.

**Conclusions:**

Patients with end-stage renal disease, and hemodialysis patients in particular, have greatly increased risk of *Staphylococcus aureus* bacteremia compared to population controls. Future challenges will be to develop strategies to reduce *Staphylococcus aureus* bacteremia-related morbidity and death in this high-risk population.

**Electronic supplementary material:**

The online version of this article (doi:10.1186/s12879-014-0740-8) contains supplementary material, which is available to authorized users.

## Background

Bacteremia in hemodialysis and renal transplant patients is most commonly caused by *Staphylococcus aureus* (SA) [1-6]. SA is also the leading cause of peritoneal dialysis catheter exit-site infections and is also frequently the cause of peritoneal dialysis-related peritonitis [7]. In addition, SA bacteremia (SAB) is associated with severe complications such as endocarditis, osteomyelitis, pneumonia, and meningitis – conditions that are often fatal despite relevant antibiotic treatment [8-12].

Use of intravascular catheters, fluid overload, accumulation of dialysis fluid in the abdomen affecting the lung volume, and the negative impact of the uremic state on immune function, are all potential factors for bacteremia among dialysis patients [13,14]. In renal transplant recipients, immunosuppressive therapy as well as frequent urinary tract infections may increase the risk of SAB [15]. Although SAB is relatively common among end-stage renal disease (ESRD) patients, case fatality in patients with SAB as well as risk factors associated with SAB are not well characterized.

The objectives of this study were to: 1) investigate and compare the incidence of SAB among ESRD patients to that of population controls in Denmark; 2) estimate the risk of SAB according to renal replacement therapy (RRT) (dialysis patients and renal transplant recipients); 3) identify risk factors for SAB within the ESRD population; and 4) estimate the case fatality rate following SAB.

## Methods

### Study design and population

We performed a nationwide, population-based cohort study among ESRD patients and matched population controls in the period 1 January 1992 to 31 December 2009. This study was facilitated by the prospective registration of ESRD patients in Denmark that has been available in the Danish Nephrology Registry (DNR) since 1 January 1990 [16].

We defined patients with ESRD as patients who had been on continuous dialysis for at least 90 days or patients with a renal graft. The estimated prevalence of ESRD in Denmark is 0.08% [16]. ESRD patients who were at least 16 years of age and had no prior recorded SAB episode at the time of ESRD diagnosis were included in the study. For ESRD patients the follow-up period were stratified according to the type of renal replacement therapy: peritoneal dialysis, hemodialysis, or renal transplantation. Patients whose type of renal replacement therapy was altered during the study period (e.g. from hemodialysis to renal transplantation) could contribute risk time to more than one of the subgroups.

### Population controls

Individuals with no diagnosis of ESRD were sampled from the Danish Civil Registration System (CRS) and used for comparative analyses. To allow for comparisons of rare events, we identified for each ESRD patient 19 population controls matched on gender and age (month and year of birth) on the day the corresponding patient was diagnosed with ESRD.

### Data sources

For this study, we linked five nationwide databases using the unique person identifier assigned by the Danish Civil Registration System: the Danish Nephrology Registry (DNR), the Danish Civil Registration System (CRS), the Danish National Patient Registry (DNPR), the Danish Registry of Causes of Death (DRCD), and the Danish Staphylococcal Bacteremia Database.

#### The Danish Nephrology Registry (DNR)

All nephrology departments in Denmark that provide care for patients with ESRD have been required to report clinical and treatment data identifiable by CRS number to the DNR database since 1 January 1990 [16]. Treatment of patients with ESRD is centralized to 15 Departments of Nephrology, four of which had status as transplantation centres during the study period.

#### The Danish Civil Registration System (CRS)

The CRS, a national registry of all Danish residents, was used to obtain information on date of birth, sex, date of emigration, and date of death. Through CRS, multiple registrations of the same patient were prevented as all individuals are assigned a unique person identifier, which allows accurate linkage across all the five registries [17].

#### The Danish National Patient Registry (DNPR)

The DNPR has collected nationwide data on all hospital admissions since 1977. For each hospitalization, the DNPR records the personal identification number (CRS number), hospital department involved, discharge diagnoses, and dates of admission and discharge. From 1977 to 1993, diagnosis codes were coded with reference to the 8^th^ revision of the International Classification of Diseases (ICD-8); since 1994, they have been coded with reference to the 10^th^ revision (ICD-10). The treating physician registered the final diagnosis codes at the time of discharge.

#### The Danish National Diabetes Register (DNDR)

The DNDR contains nationwide information about diabetic patients in Denmark.

The registry is based on data from the DNPR, the National Health Insurance Service Registry, the Register of Medical Product Statistics, and the CRS. Main variables include: civil registration, gender, residence, date and cause of inclusion. Data have been collected from 1996 onwards [18].

#### The Danish staphylococcal bacteremia database

Registration of SAB cases has been carried out since 1956 at the Staphylococcal Laboratory at the Statens Serum Institut (SSI), Copenhagen. The Staphylococcal Laboratory receives blood culture isolates from >95% cases of SAB identified by the Departments of Clinical Microbiology in Denmark for typing and national surveillance of antimicrobial susceptibility. All samples for SAB were required on clinical indication. No screening for SAB was performed during the study period. The database has been described in detail elsewhere [9,10,19]. We included the following data from this database in our study: the date of the first blood culture positive for *Staphylococcus aureus*, methicillin susceptibility of the isolated strain, and origin of bacteremia (hospital associated or community acquired). Hospital-acquired SAB was defined as SAB diagnosed more than 48 hours after admission or catheter-related infections. Health care-associated SAB included individuals in regular hemodialysis or regular intravenous infusions of chemotherapy or antivirals. Community acquired SAB was defined as SAB diagnosed <48 hours after hospital admission with none of the above-mentioned health-care related exposures. If a patient had recurrent SABs in the study period, only the first episode was included in our study to eliminate bias by multiple SAB episodes occurring in highly susceptible individuals.

### Data on comorbidity

The CCI includes 19 major disease categories and has been adapted and validated for use with hospital discharge data in ICD databases. The CCI is considered a reliable method for measuring comorbidity in clinical research [20]. In this study, we calculated a modified Charlson comorbidity index (m-CCI) score for each study participant based on the complete hospital discharge history. The m-CCI score did not include renal disease or diabetes mellitus since both variables were included in the analyses as independent covariates.

### Statistical analyses

#### Time at risk

ESRD patients were observed from the date of initiating RRT. Population controls entered the study on the same day as their matched ESRD patient. We determined time at risk from the date of first observation until the date of death, emigration, first SAB, or 31 December 2009, whichever came first.

#### Incidence rate (IR) and incidence rate-ratio (IRR)

The IR of first-time SAB was calculated for ESRD patients and compared to the IR for population controls. Poisson regression analysis was used to determine IRs and IRRs.

#### Risk factors

Cox regression was used to determine hazard ratios and identify risk factors for the first episode of SAB in ESRD patients. The following variables were entered into the model: gender, cause of ESRD (glomerulonephritis, diabetes (DM) type I and II, chronic interstitial nephritis (CIN), hypertensive kidney disease, polycystic kidney disease, nephrosclerosis, and vasculitis), and the following time-varying covariates (TVCs): age (<50 years, 50–59 years, and ≥60 years), replacement therapy (transplantation, hemodialysis, and peritoneal dialysis), and calendar period (1992–1996, 1997–2001, 2002–2006, and 2007–2009). Comorbidity as assessed by the m-CCI score was calculated for each patient on the date of their study entry. Three comorbidity levels were defined according to the m-CCI score: 0 = low, 1–2 = medium, and ≥3 = high.

#### Origin of SAB and methicillin resistance

The origin of incident SAB cases was tabulated (hospital associated or community acquired) as well as the susceptibility of the isolated strains to methicillin (resistant, sensitive, or unknown).

#### Case fatality rates following SAB

We computed Kaplan-Meier estimates for the 30- and 90-day case fatality rate of patients according to RRT and their matched population controls. The resulting survival curves were examined for differences by log-rank test. We used Stata software, version 12.0 (StataCorp, College Station, TX, USA) for statistical analyses. The study was approved by the Danish Data Protection Agency (record no. 2010-41-4935) and the Danish Health and Medicines Agency (record no. 2010-331-0462).

## Results

The study population encompassed 10,908 ESRD patients and 189,850 population controls, providing 39,478 and 1,338,339 person-years of follow-up (PYFU), respectively. Median years of follow-up were 2.3 (IQR: 0.8–5.1) among ESRD patients and 6.4 (IQR: 2.9–10.5) among population controls (Table 1). We identified a total of 1,408 first episodes of SAB in ESRD patients and 733 first episodes of SAB in population controls.

**Table 1:** **Characteristics of patients with end-stage renal disease and population control at study entry**

**Variable**	**ESRD individuals (N = 10,908)**	**Population controls (N = 189,850)**
**Median years of follow-up, (interquartile range)**	2.3 (0.8–5.1)	6.4 (2.9–10.5)
**Age at entry (N = 200,758)**		
16-49	2,481 (22.7%)	39,901 (21.0%)
50-64	3,314 (30.4%)	57,471 (30.3%)
65+	5,113 (46.9%)	92,478 (48.7%)
**Sex (N = 200,758)**		
Female	4,059 (37.2%)	71,664 (37,7%)
Male	6,849 (62.8%)	118,186 (62.3%)
**Diabetes (N = 200,758)**		
No	8,443 (77.4%)	180,475 (95.1%)
Yes	2,465 (22.6%)	9,375 (4.9%)
**m-CCI score (N = 200,758)**		
Low	3,145 (28.8%)	133,599 (70.4%)
Moderate	4,232 (38.8%)	43,923 (23.14%)
High	3,531 (32.4%)	12,328 (6.5%)
**SAB event (N = 200,758)**		
No	9,500 (87.1%)	189,117 (99.6%)
Yes	1,408^1^ (12.9%)	733 (0.4%)

ESRD: end-stage renal disease; m-CCI: modified Charlson Comorbidity Index; SAB: *Staphylococcal aureus* bacteremia.

**NOTE.** Baseline characteristics. Data are no. of individuals, unless otherwise indicated.

^1^Distribution of SAB events according to type of renal replacement therapy: renal transplantation = 23 events, hemodialysis = 1,069 events, and peritoneal dialysis = 316 events.

### Incidence rates and relative risk of SAB

The overall IR of first-time SAB was 35.7 per 1,000 PYFU (95% confidence interval (CI), 33.8–37.6) for ESRD patients and 0.5 per 1,000 PYFU (95% CI, 0.5–0.6) for population controls, yielding an unadjusted incidence rate-ratio [21] of 65.1, (95% CI, 59.6–71.2). When controlling for potential confounders including sex, age, diabetes mellitus, and m-CCI, we found an adjusted IRR of 28.6 (95% CI, 23.3–35.3) for first-time SAB comparing ESRD patients with population controls. Stratified by mode of RRT, the IR of SAB was 46.9 (95% CI, 44.1–49.8) per 1,000 PYFU for patients in hemodialysis, 22.2 (95% CI, 19.8–24.8) per 1,000 PYFU for patients in peritoneal dialysis, and 9.4 (95% CI, 6.0–14.2) per 1,000 PYFU for renal transplant recipients. The corresponding unadjusted incidence rate-ratios comparing ESRD patients with their respective population controls were 78.3 (95% CI, 70.5–86.9) for hemodialysis patients, 77.1 (95% CI, 31.4–189) for transplant recipients, and 44.9 (95% CI, 37.7–53.4) for peritoneal dialysis patients.

The 1-year risk of SAB was 8.0% (95% CI, 7.5–8.5) for ESRD patients and 0.05% (95% CI, 0.04–0.06) for population controls. The corresponding 5-year risk of SAB was 16.1% (95% CI, 15.2–17.0) for ESRD patients and 0.25% (95% CI, 0.23–0.28) for population controls.

**Figure 1:**
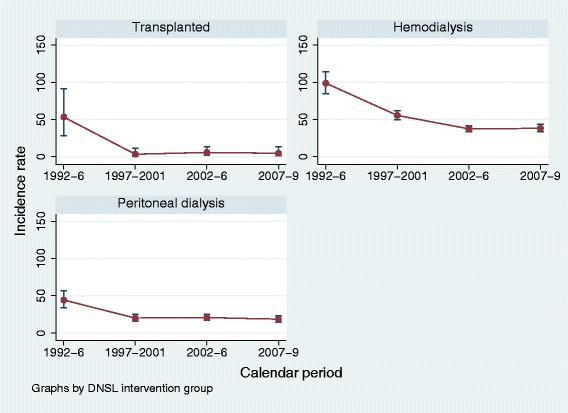
**Incidence rates stratified by renal replacement therapy during the study period from 1992–2009.**

Figure 1 shows the association between calendar time and risk of SAB over the entire study period (year 1992–2009) for ESRD patients and population controls. The risk of SAB decreased among ESRD patients regardless of RRT mode as seen in Additional file 1. The risk of SAB among population controls increased slightly from an IR of 0.3 per 1,000 PYFU (95% CI, 0.2–0.4) in the first calendar period (years 1992–1996) to an IR of 0.6 per 1,000 PYFU (95% CI, 0.6–0.7) in the last calendar period (years 2007–2009).

### Risk factors among persons with ESRD

Table 2 illustrates potential risk factors for a first-time SAB among ESRD patients. In the analysis we adjusted for sex, age, diabetes, m-CCI, calendar period, cause of ESRD, and type of RRT. Factors associated with a significantly increased risk of SAB were high vs. low m-CCI score (HR = 1.6 [95% CI, 1.4–1.9]), hemodialysis (HR = 5.4 [95% CI, 4.1–7.1]), and peritoneal dialysis (HR = 2.1 [95% CI, 1.5–2.9]) compared to renal transplantation as mode of RRT. Moreover, the risk of SAB declined during the study period.

**Table 2:** **Hazard Ratios for first** ***Staphylococcus aureus*** **bacteremia among patients with end-stage renal disease according to potential risk factors**

	**Unadjusted**	Adjusted*	
**Characteristic**	**HR (95% CI)**	**HR (95% CI)**	***P*** **value (in adjusted analysis)**
**Sex**			
Female	1 (Reference)	1 (Reference)	
Male	1.12 (1.00–1.24)	1.08 (0.96–1.20)	0.20
**Age**			
<50 years	1 (Reference)	1 (Reference)	
50-64 years	1.02 (0.90–1.16)	0.87 (0.76–1.00)	0.06
≥65 years	1.09 (0.96–1.25)	0.87 (0.75–1.00)	0.06
**Diabetes mellitus**			
No	1 (Reference)	1 (Reference)	
Yes	1.33 (1.18–1.50)	1.05 (0.91–1.22)	0.52
**m-CCI**			
Low	1 (Reference)	1 (Reference)	
Moderate	1.33 (1.15–1.54)	1.20 (1.03–1.40)	0.02
High	1.86 (1.62–2.14)	1.61 (1.37–1.91)	<0.001
**Calendar period**			
1992–1996	1 (Reference)	1 (Reference)	
1997–2001	0.92 (0.80–1.06)	0.86 (0.75–0.98)	0.03
2002–2006	0.81 (0.70–0.93)	0.72 (0.62–0.83)	<0.001
2007–2009	0.78 (0.64–0.94)	0.69 (0.57–0.84)	<0.001
**Cause of ESRD**			
Glomerulonephritis	1 (Reference)	1 (Reference)	
Diabetes mellitus	1.55 (1.29–1.86)	1.77 (0.95–1.45)	0.13
CIN	1.05 (0.84–1.31)	0.98 (0.78–1.22)	0.85
Hypertensive kidney	1.24 (1.04–1.48)	1.15 (0.96–1.38)	0.12
Polycystic kidney	0.55 (0.41–0.74)	0.54 (0.40–0.72)	<0.001
Vasculitis	1.42 (1.06–1.90)	1.25 (0.92–1.68)	0.15
Other	1.19 (0.94–1.52)	1.09 (0.85–1.40)	0.50
**Renal replacement therapy**			
Transplantation	1 (Reference)	1 (Reference)	
Hemodialysis	3.95 (3.34–4.68)	5.41 (4.13–7.09)	<0.001
Peritoneal dialysis	0.58 (0.51–0.65)	2.07 (1.46–2.95)	<0.001

*Adjusted for sex, age, diabetes, modified Charlson Comorbidity Index, calendar period, cause of ESRD, and renal replacement therapy. m-CCI score categories low = 0-1, moderate = 2-3, and high > 3. HR: hazard ratio; CI: confidence interval; m-CCI: modified Charlson Comorbidity Index; ESRD: end-stage renal disease.

### Characteristics of SAB

Origin of SAB – hospital associated or community acquired-among ESRD patients are shown in Table 3. Where the frequency of hospital associated-SAB infections among ESRD patients was high (67.2%), the overall proportion of methicillin-resistant SA (MRSA) was low. Only 8 (0.7%) of the 995 tested strains were caused by MRSA in the entire study population (6 among ESRD patients and 2 among population controls).

**Table 3:** **Characteristics of patients with** ***Staphylococcal aureus*** **bacteremia**

**Characteristics**	**ESRD**	**Population controls**
**Origin of infection**		
Community acquired	60 (5.08%)	193 (33.6%)
Hospital associated	794 (67.2%)	331 (57.7%)
Unknown	328 (27.8%)	50 (8.7%)
**Methicillin category**		
Methicillin sensitive	815 (99.3%)	272 (99.3%)
Methicillin resistant	6 (0.7%)	2 (0.7%)
**Complications**		
None/not registered	717 (93.1%)	224 (84.5%)
Endocarditis	24 (3.1%)	15 (5.7%)
Osteomyelitis	23 (3.0%)	24 (9.1%)
Meningitis*	6 (0.8%)	2 (0.6%)

**NOTE.** No. of individuals (%). ESRD: end-stage renal disease *from 1990.

Frequent complications following SAB were endocarditis and osteomyelitis. Endocarditis occurred in 3.1% among ESRD patients and in 5.7% among population controls. Osteomyelitis occurred in 3.0% among ESRD patients and in 9.1% among population controls.

### Case fatality following SAB

**Figure 2:**
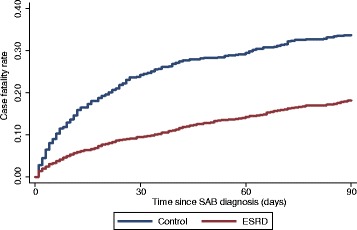
**90-day case fatality following SAB in ESRD patients and population controls.**

The case fatality following SAB for ESRD patients and population controls is shown in Figure 2. In ESRD patients, the 30-day case fatality rate after a diagnosis of SAB was 9.4% (95% CI, 8.0–11.1) compared with 24.2% (95% CI, 21.2–27.6) among population controls. The 90-day case fatality rate among ESRD patients was 18.2% (95% CI, 16.2–20.3) compared with 33.7% (95% CI, 30.3–37.3) among population controls. Stratified by RRT, the 30-day case fatality rate was 7.4% (95% CI, 5.0–10.9) among patients receiving peritoneal dialysis, 10.2% (95% CI, 8.5–12.1) among patients receiving hemodialysis, and 4.4% (95% CI, 0.6–27.1) among renal transplant recipients. Ninety-day case fatality rates were 14.5% (95% CI, 11.0–18.9) among patients receiving peritoneal dialysis, 19.5% (95% CI, 17.3–22.1) among patients receiving hemodialysis, and 4.4% (95% CI, 0.6–27.1) among renal transplant recipients.

## Discussion

The present study is the first to investigate, on a national level, the relative risk of SAB among ESRD patients receiving RRT. In our study we found the risk of first-time SAB to be 65-fold higher among ESRD patients compared to population controls. Even after adjusting for sex, age, diabetes mellitus, and m-CCI the risk was 28-fold higher in ESRD patients compared to population controls. Hemodialysis patients in particular had a greatly increased risk of SAB. Nevertheless, the incidence of SAB declined throughout the study period possibly indicating enhanced compliance with infection control precautions by both patients and staff. The reasons for this decline are unknown and could be do to changes in the virulence or other genetic attributes in circulating strains of *Staphylococcus aureus*. The reasons for this decline are unknown and could be do to changes in the virulence or other genetic attributes in circulating strains of *Staphylococcus aureus* [22]. In addition, we identified characteristics, e.g. high m-CCI score and hemodialysis among ESRD patients that may be selectively targeted for interventions to further reduce the risk of SAB.

The strengths of our study include the use of population-based, nationwide cohorts with inclusion of all adults receiving RRT (transplant recipients, and patients receiving hemodialysis or peritoneal dialysis) in an entire country, minimal loss to follow-up, and the availability of comprehensive hospitalization data and microbiological data on each SAB case. The m-CCI enabled us to adjust for underlying diseases, and the large study size provided statistical precision for the estimates. Because we only included the first blood cultures positive for *Staphylococcus aureus* in the analyses, our estimates were not biased by multiple SAB episodes occurring in highly susceptible individuals.

A number of factors may influence the increased risk of SAB in ESRD patients that we observed in this study. As reported in previous studies [2,23], we found that older age, male gender and underlying comorbid medical conditions increased the risk of SAB. Furthermore a wider use of immunosuppressive therapy among ESRD patients may contribute to a higher risk of SAB [9].

We found that SAB was hospital associated in 67.2% of cases among ESRD patients and 57.7% among population controls. Previous results have documented that the incidences of both hospital associated and community acquired SAB have been increasing [9]. In Denmark, compared to hospital acquired SAB, community acquired SAB has increased relatively more and has a higher case fatality rate among adults [9]. Increased case fatality among population controls compared with ESRD patients can be explained by various reasons including; early diagnose of SAB among ESRD patients, especially hemodialysis patients, who visits the dialysis clinics several times during a week, increased awareness of feverish disease in ESRD patients, and the fact that dialysis catheter exit-site infections are the cause of a high amount of the acquired infections, which thereby have a better prognosis because of immediate catheter removal. Furthermore, previous studies have found higher rates of complications among persons with community acquired SAB compared to hospital acquired SAB which also may explain the relatively high case fatality rate among the population with SAB in our study [24,25].

We observed low rates of MRSA in the study population, which correlates well with previous findings [4,9,23]. However, the incidence of MRSA infections in the general population has increased in Denmark during the later years [26], i.e. after the end of our study period (2009).

Some limitations of this study may have impacted our findings. Physicians may have a lower threshold for hospital admission of ESRD and for ordering blood cultures in patients with signs and symptoms compatible with SAB than for patients who did not undergo RRT, which could cause us to overestimate the relative risk of SAB in ESRD patients. In fact, we found a lower case fatality rate among ESRD patients compared to population controls. This may indicate that, for some ESRD patients, the severity and complications of SAB upon hospital admission are less than in the matched population controls – possibly due to more closely monitoring for blood stream infection. A severity index score could not be calculated from this dataset, thus changes in disease severity during the study period could not be assessed or analysed. Furthermore, we only included cases with SA positive blood culture; hence the true incidence of invasive SA disease may have been underestimated. Also, coding errors may occur in routine hospital discharge data, leading to misclassifications in both the ESRD group and population control group. Furthermore, we did not have any data on type of dialysis access. Our data does not reflect the total burden of SAB in the study population (since only first episodes of SAB were included). Finally, we had no information on immunosuppressive regimens and could not assess the effect of individual drugs on SAB. According to other studies [15] risk of infection is not significantly different for specific immunosuppressive drug regimens.

## Conclusions

In conclusion, patients receiving RRT for ESRD, and hemodialysis patients in particular, have a greatly increased risk of SAB compared to population controls. SAB was associated with significant case fatality both in ESRD patients and in population controls. As there are currently no prophylactic vaccines for SA, future challenges will be to develop other preventive measures and treatment strategies to reduce morbidity and case fatality from SAB in high-risk populations such as ESRD patients in general, and hemodialysis patients in particular.

## Additional file

Additional file 1:
**Incidence rate of first episode of SAB according to type of renal replacement therapy.** Additional table showing incidence rates and incidence rate ratios after first episode of *Staphylococcus aureus* bacteremia according to type of renal replacement therapy; renal transplant recipients, hemodialysis patients or peritoneal dialysis patients.
