# Supplementary material for: Risk and prognosis of Staphylococcus aureus bacteremia among individuals with and without end-stage renal disease: a Danish, population-based cohort study
Source: BMC Infect Dis. 2015 Jan 8;15:6. doi: 10.1186/s12879-014-0740-8 (PMC4296555; doi:10.1186/s12879-014-0740-8)
Supplement: Additional file 1: — Incidence rate of first episode of SAB according to type of renal replacement therapy. Additional table showing incidence rates and incidence rate ratios after first episode of Staphylococcus aureus bacteremia according to type of renal replacement therapy; renal transplant recipients, hemodialysis patients or peritoneal dialysis patients. [file 12879_2014_740_MOESM1_ESM.pdf]

Additional file 1. Incidence rate of first episode of SAB according to type of renal replacement therapy

	Incidence rate		Incidence rate ratio
**Renal replacement therapy mode**	**First calendar period (year 1992-1996)**	**Last calendar period (year 2007-2009)**	**First vs last calendar period**
Transplant recipients	53.2[1] (28.3–90.9)	4.5[1] (0.9–13.1)	0.08 (0.02-0.30)
Hemodialysis patients	98.8[1] (84.4–114.4)	38.1[1] (33.5–43.2)	0.39 (0.32-0.47)
Peritoneal dialysis patients	44.2[1] (34.0-56.6)	18.4[1] (14.5–23.1)	0.42 (0.30-0.58)

NOTE.

[1]Per 1,000 person years follow up
